# Performance characteristics of a quantitative PCR assay on repository stool specimens and smeared filter-paper cards

**DOI:** 10.1186/s13104-020-05340-7

**Published:** 2020-10-30

**Authors:** Michele D. Tisdale, Indrani Mitra, Andrea J. McCoy, Mark P. Simons, Nathanael D. Reynolds, Brett E. Swierczewski, Jie Liu, Eric R. Houpt, Jamie A. Fraser, Mark S. Riddle, David R. Tribble, Tahaniyat Lalani

**Affiliations:** 1 Infectious Disease Clinical Research Program, Department of Preventive Medicine and Biostatistics, Uniformed Services University of the Health Sciences, Bethesda, MD USA; 2 Naval Medical Center Portsmouth, Portsmouth, VA USA; 3 The Henry M Jackson Foundation for the Advancement of Military Medicine, Inc, Bethesda, MD USA; 4 Naval Medical Research Unit-6, Lima, Peru; 5 Armed Forces Research Institute of Medical Sciences, Bangkok, Thailand; 6 University of Virginia, Charlottesville, VA USA; 7 Department of Preventive Medicine and Biostatistics, Uniformed Services University of the Health Sciences, Bethesda, MD USA; 8 Infectious Disease & Travel Clinic, Building 3, 1st Floor, Naval Medical Center Portsmouth, Portsmouth, VA 23708 USA

**Keywords:** Diarrhea, Polymerase chain reaction, Travel, Archives

## Abstract

**Objective:**

Stool repositories are a valuable resource for retrospective analyses including quantitative PCR assays to distinguish between asymptomatic shedding and clinical disease. The suitability of archival specimens for this purpose is unclear and requires assessment. We conducted a pilot study to evaluate pathogen detection by TaqMan Array Card (TAC) in travelers’ diarrhea (TD) stool specimens stored for 1–13 years, as well as the impact of transporting specimens on Whatman FTA Elute cards (FTA Cards) on detection.

**Results:**

The positive percent agreement (PPA) for TAC on stool vs. microbiologic testing was lower than our a priori PPA estimate of 80% for most pathogens: *Shigella* spp. (100% [95%CI 69–100%]), enterotoxigenic *E coli* (ETEC) (63% [95%CI 49–75%]), *Campylobacter* spp. (66% [95%CI 43–85%]) and Norovirus (37% [95%CI 16–61%]). Use of the FTA card resulted in a further reduction of PPA. Our findings suggest that archival specimens may lead to insensitive detection on quantitative PCR assays due to degradation of nucleic acid with prolonged storage, although our limited sample size precluded us from evaluating the impact of storage duration on nucleic acid yield. Additional studies are needed to understand the impact of storage duration on quantitative PCR data.

## Introduction

Polymerase Chain Reaction (PCR) assays have significantly improved the detection of travelers’ diarrhea (TD) pathogens but interpreting results is often challenging due to asymptomatic shedding and multi-pathogen detection [1–3]. Studies evaluating the performance characteristics of PCR assays have largely been conducted in clinic or hospital settings using diarrheal samples tested within days of collection [4, 5]. This sampling method is limited by the infrequency of TD pathogens such as enterotoxigenic *Escherichia coli, Shigella* spp. and *Campylobacter* spp. and the lack of appropriately matched control specimens (e.g. asymptomatic travelers) for attribution of TD to detected pathogens. PCR data from longitudinal pediatric cohorts in developing countries cannot be readily extrapolated to adult TD populations due to differences in the host and environment that impact pathogen load and multi-pathogen detection in stool samples [6].

Biorepositories of clinically characterized diarrheal and non-diarrheal specimens provide an alternative resource to investigate clinical interpretation of PCR assay results. Biorepository specimens could be used to determine the odds of TD associated with detection of specific pathogens, and potentially refine estimates using quantification cycle (Cq) thresholds for pathogens detected in cases and controls. The suitability of archival specimens for DNA/RNA amplification and impact on assay performance is unclear, due to potential degradation. There are also logistical challenges in transporting frozen fecal specimens from global biorepositories to a single testing site while maintaining the cold-chain, which can be cost-prohibitive. The Whatman FTA Elute Card^®^ (FTA card, GE Healthcare Life Sciences, WB120411, Marlborough, MA, USA) is an attractive alternative to conventional storage and shipment methods, as they can be shipped by regular mail at room temperature. The impact of using FTA Cards for sample storage and transportation on PCR sensitivity has not been evaluated.

## Main text

### Methods

We conducted a pilot study using a customized TD TaqMan Array Card (TAC, Life Technologies, Carlsbad, CA, USA) to assess the feasibility of using archived diarrheal specimens, of varying time periods, focusing on four TD pathogens: enterotoxigenic *E coli* (ETEC), *Shigella* spp., *Campylobacter* spp., and Norovirus. Two United States Department of Defense (DoD) fecal repositories of adult TD cases, tested using standard microbiologic methods at the time of sample collection, were utilized for the study [7, 8]:

- Naval Medical Research Unit-6, Lima, Peru (NAMRU-6): Archived samples collected between 2003 and 2010 were tested for Campylobacter and Shigella by stool culture, Norovirus by PCR, and ETEC by stool culture followed by PCR of 5 colonies. Samples collected in 2013 were tested for all 4 pathogens by PCR.
- Armed Forces Research Institute of Medical Sciences (AFRIMS), Bangkok, Thailand: Archived samples collected between 2013 and 2016 were tested by PCR (Luminex xTAG Gastrointestinal Pathogen Panel), stool culture and ELISA. A positive result from any test was considered positive for the pathogen.

We evaluated the Positive Percent Agreement (PPA) and Negative Percent Agreement (NPA) of TAC for detecting TD pathogens in archived fecal specimens, using results from previous microbiologic testing as the ‘benchmark’. We also evaluated the impact of storage duration and use of FTA cards on TAC sensitivity. Prior reports estimated a sensitivity and specificity of > 90% of PCR assays albeit with shorter sample storage duration (4, 5). We estimated a PPA and NPA of 80% for the TAC assay and a precision of 10%. Approximately 65 positive stool specimens per pathogen for ETEC, *Shigella* spp., *Campylobacter* spp., and Norovirus and 30 samples negative for all pathogens, were requested. Descriptive statistics were used to evaluate the PPA, NPA and 95% confidence intervals (CI) and the Mann–Whitney *U* was performed for continuous variables. The study was approved by the Institutional Review Boards (IRB) of the Uniformed Services University and reviewed by the research office at NAMRU-6 and Walter Reed Army Institute of Research/Armed Forces Research Institute, Thailand (AFRIMS).

**Fig. 1:**
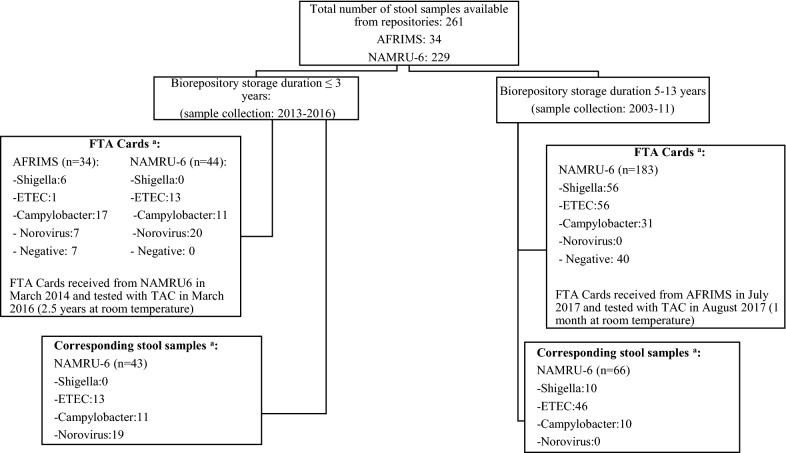
Flow diagram of samples received from both repositories and duration of storage prior to testing

All stool samples were thawed, vortexed and approximately 20µL smeared onto an FTA card and shipped at room temperature to Naval Medical Center Portsmouth (NMCP) for testing. Due to logistic and regulatory constraints a limited number of stool specimens were shipped from NAMRU-6 and only smeared FTA Cards were shipped from AFRIMS. 109 out of 261 (41.7%) stool samples were aliquoted and shipped at –20 °C for testing (Fig. 1). Laboratory personnel performing TAC testing were blinded to the results from previous testing. FTA Cards from NAMRU-6 were received as a single batch two years before stool samples due to delays in getting required approvals for shipment (Fig. 1). FTA cards were stored at room temperature (20–24 °C) until stool samples were received. Stool samples were tested within a month of receipt from NAMRU-6 and to reduce between run variability, we included corresponding FTA Card and stool samples in the same PCR run. FTA Cards from AFRIMS were batch shipped to NMCP and tested within a month of receipt. Extraction and TAC testing was performed as previously described [9].

### Results

Two hundred and sixty-one samples for targeted pathogens were available: ETEC (n = 70), Shigella (n = 62), Campylobacter (n = 59), Norovirus (n = 27). Four samples were positive for more than 1 pathogen and 47 samples were negative for all targeted pathogens.

The PPA for TAC on stool vs. benchmark was lower than our a priori PPA estimate of 80% across all pathogens except Shigella (Table 1). For example, the ETEC TAC PPA was 62.7% [95%CI 49.2–75.0]. The ETEC TAC PPA was numerically lower in samples stored for ≤ 3 years vs. those stored for 5–13 years (69.2% [95%CI 38.6–90.9] vs. 60.8% [95%CI 45.4–74.9]) although this was confounded by the use of direct-on-stool PCR as benchmark for samples stored for ≤ 3 years and culture followed by PCR of colonies in samples stored for 5–13 years. The low PPA contrasts with data published by Liu et al. reporting 100% [95%CI 69.2–100.0] TAC PPA on 10 ETEC positive samples stored for 2–4 years, using stool culture and PCR of colonies as the benchmark [10]. Of note, the 95% CI in both studies is large due to the small sample size. Similarly, TAC PPA for 21 Campylobacter positive stool samples (66.6% [95%CI 43.0–85.4]) was lower than the published estimate of 97.1% [95%CI 85.1–99.9] [4]. Use of the FTA card resulted in a further reduction of PPA across bacterial targets (Table 1). A large proportion of samples with discordant results (i.e. TAC positive stool and TAC negative FTA card) had high stool Cq values (between 30 and 35). Of the 13 paired samples with discordant ETEC detection, 11 had stool Cq values between 30 and 35. A similar proportion of discordant samples was observed for Campylobacter (15/17) but not for Shigella (4/9) or Norovirus (6/18). TAC PPA for Norovirus was significantly lower than bacterial targets in stool (36.8% [95%CI 16.2–61.6]) and FTA Card (51.8% [95%CI 32.0–71.3]). TAC detected an additional 77 and 50 samples positive for targeted pathogens on stool and FTA card respectively, compared to benchmark testing. A decrease in PPA with increasing duration of storage (i.e. ≤ 3 years and 5–13 years) was noted for ETEC and Campylobacter positive FTA Cards, although the analysis was limited by small sample size and wide confidence intervals of PPA estimates between strata of storage duration and could not be assessed for Shigella or Norovirus (Table 1). To evaluate whether the sensitivity loss in samples with prolonged storage duration was due to PCR inhibition, we compared the difference in extrinsic control Cq between the sample and corresponding extraction blank. A higher Cq value in stool or FTA card compared to the corresponding extraction blank indicated a loss in PCR signal due to inhibition. A Cq difference of 3.3 was considered equivalent to a 1-log loss in PCR signal due to inhibition. The proportion of samples that met this criterion were similar in the two storage duration strata, suggesting that loss of sensitivity was not due to lower extraction/amplification efficiency or inhibition.

**Table 1:** Summary of performance characteristics of PCR assay tested in this study and reported literature

TAC on stool vs. benchmark				
Pathogen	This study—total number of stool samples tested by TAC = 109		Reported literature	
	Median storage duration (IQR) and no. of samples:	PPA and NPA	Study design	PPA and NPA
ETEC LT (labile toxin) ST (stable toxin)	No of samples: 59 11.5 (7.2-12.0) years ≤ 3 years: 13 samples 5–13 years: 46 samples	PPA:62.7 (49.2-75.0) NPA:70.0 (55.4-82.1) Stratified by duration: ≤ 3 years: PPA: 69.2 (38.6–90.9) NPA: 66.7 (47.2–82.7) 5–13 years: PPA: 60.8 (45.4–74.9) NPA: 75.0 (50.9–91.3)	Sample size: 109 (10 ETEC positive) Comparator: Culture + PCR of 5 colonies PCR platform: TAC Storage duration: 2–4 years (10)	PPA:100.0 (69.2–100.0) NPA (ETEC LT): 55.0 (41.6–67.9) NPA (ETEC ST): 69.7 (57.1–80.4)
Campylobacter	No of samples:21 2.6 (2.6-11.4) years ≤ 3 years: 11 5-13 years: 10	PPA:66.6 (43.0–85.4) NPA:69.3 (58.6–78.7) Stratified by duration: ≤ 3 years: PPA: 63.6 (30.8–89.0) NPA: 68.8 (50.0–83.8) 5–13 years: PPA: 70.0 (34.8–93.3) NPA: 69.6 (56.0–81.2)	Sample size: 1557 (35 Campylobacter positive) Comparator: Culture PCR platform: BioFire FilmArray GI panel Storage duration: days-weeks (4)	PPA:97.1 (85.1–99.9) NPA:98.4 (97.7–99.0)
Shigella/EIEC	No of samples:10 7.6 (6.7–10) years 5–13 years: 10	PPA:100.0 (69.2-100.0) NPA:82.8 (74.0–89.6)	Sample size: 1557 (49 Shigella/EIEC positive) Comparator: Culture (PCR for EIEC) PCR platform: BioFire FilmArray GI panel Storage duration: days-weeks (4)	PPA:95.9 (86.0–99.5) NPA:98.5 (99.5–130.0)
Norovirus	No of samples:19 2.6 (2.6–2.6) years ≤ 3 years: 19	PPA:36.8 (16.2–61.6) NPA:80.0 (70.2–87.6)	Sample size: 1557 (55 Norovirus positive) Comparator: RT-PCR PCR platform: BioFire FilmArray GI panel Storage duration: days-weeks (4)	PPA:94.5 (84.9–98.9) NPA:98.8 (98.1–99.3)

TAC on FTA card vs. standard microbiology (benchmark) This study – total number of FTA cards tested by TAC = 261			
Pathogen	Median storage duration (IQR) and no. of samples:	PPA and NPA	Stratified by duration:
ETEC	No of samples:70; 10.5 (6.9–12.0) years ≤ 3 years: 14 5–13 years: 56	PPA:55.7 (43.3–67.6) NPA:85.3 (80.0–90.0)	≤ 3 years: PPA: 57.1 (28.8–82.3) NPA: 86.0 (75.0–93.3) 5–13 years: PPA: 55.4 (41.4–68.6) NPA: 85.0 (77.6–90.8)
Campylobacter	No of samples: 59; 10.4 (2.6–11.8) years ≤ 3 years: 28 5–13 years: 31	PPA:62.7 (49.2–75.0) NPA:82.2 (76.2–87.2)	≤ 3 years: PPA:71.4 (51.3–86.8) NPA: 84.0 (70.8–92.8) 5–13 years: PPA: 54.8 (36.0–72.6) NPA: 81.6 (74.4–87.4)
Shigella	No of samples: 62; 6.0 (2.5–7.6) years ≤ 3 years: 6 5–13 years: 56	PPA:88.7 (78.1–95.3) NPA:86.4 (81.0–91.0)	≤ 3 years: PPA: 16.6 (1.0–64.1) NPA: 94.4 (86.4–98.4) 5–13 years: PPA: 96.4 (87.6–99.6) NPA: 81.8 (74.0–88.2)
Norovirus	No. of samples: 27; 2.6 (2.6–2.6) years	PPA:51.8 (32.0–71.3) NPA:97.8 (95.0–99.3)	N/A (all samples stored ≤ 3 years)

TAC on FTA card vs. TAC on stool (benchmark)				
Pathogen	This study—total number of paired FTA cards and stool samples tested by TAC = 109		Reported Literature	
	Median storage duration (IQR) and no. of samples:	PPA and NPA	Study Design	PPA and NPA
ETEC	No. of paired samples: 52; median duration of FTA card storage: 8.8 (2.6–11.8) years ≤ 3 years: 19 5–13 years: 33	PPA: 75.0 (63.2–86.8) NPA: 91.2 (83.8–98.6) Stratified by duration: ≤ 3 years: PPA: 73.6 (48.8–90.8) NPA: 91.6 (73.0–98.9) 5–10 years: PPA: 75.8 (57.7–88.9) NPA: 90.9 (75.6–98.0)	Total sample size: 187 ETEC positive TAC on stool: 85 Storage duration: 2 years (9)	PPA:90.6 (82.2–96.0) NPA:97.6 (93.3–99.5)(3)
Campylobacter	No. of paired samples: 41; median storage duration: 7.0 (2.6–11.8) years ≤ 3 years: 17 5–10 years: 24	PPA:58.5 (42.1–73.6) NPA:88.2 (78.1–94.8) Stratified by duration: ≤ 3 years: PPA: 47.0 (22.9–72.2) NPA: 92.3 (74.8–99.0) 5-13 years: PPA: 66.6 (44.6–84.4) NPA: 85.7 (71.4–94.6)	Total sample size: 187 Campylobacter positive TAC on stool: 11 Storage duration: 2 years (9)	PPA:90.9 (58.7–99.8) NPA:100.0 (98.0–100.0)(3)
Shigella	Shigella positive TAC on stool: 27; median storage duration: 7.7 (6.0–11.3) years ≤ 3 years: 5 5–10 years: 22	PPA: 66.6 (46.0-83.4) NPA: 92.6 (84.8–97.2) Stratified by duration: ≤ 3 years: PPA: 40.0 (5.2–85.3) NPA: 94.7 (82.2–99.4) 5–13 years: PPA: 72.7 (49.8–89.2) NPA: 90.9 (78.3–97.4)	Total sample size: 187 Shigella positive TAC on stool: 18 Storage duration: 2 years (9)	PPA:88.9 (65.2-98.6) NPA:99.4 (96.8-99.9)(3)
Norovirus	No of samples:19; median duration of FTA card storage: 2.6 (2.6–2.6) years	PPA:28.0 (12.0–49.4) NPA:92.8 (85.1–97.3) Stratified by duration: ≤ 3 years: PPA: 36.4 (10.9–69.2) NPA: 81.2 (636–92.8) 5–13 years: PPA: 21.4 (4.6–50.8) NPA: 100.0 (93.2–100.0)	Total sample size: 187 Norovirus positive TAC on stool: 24 Storage duration: 2 years (9)	PPA:38.0 (18.8–60.0) NPA:100.0 (98.9–100.0)(3)

**Fig. 2:**
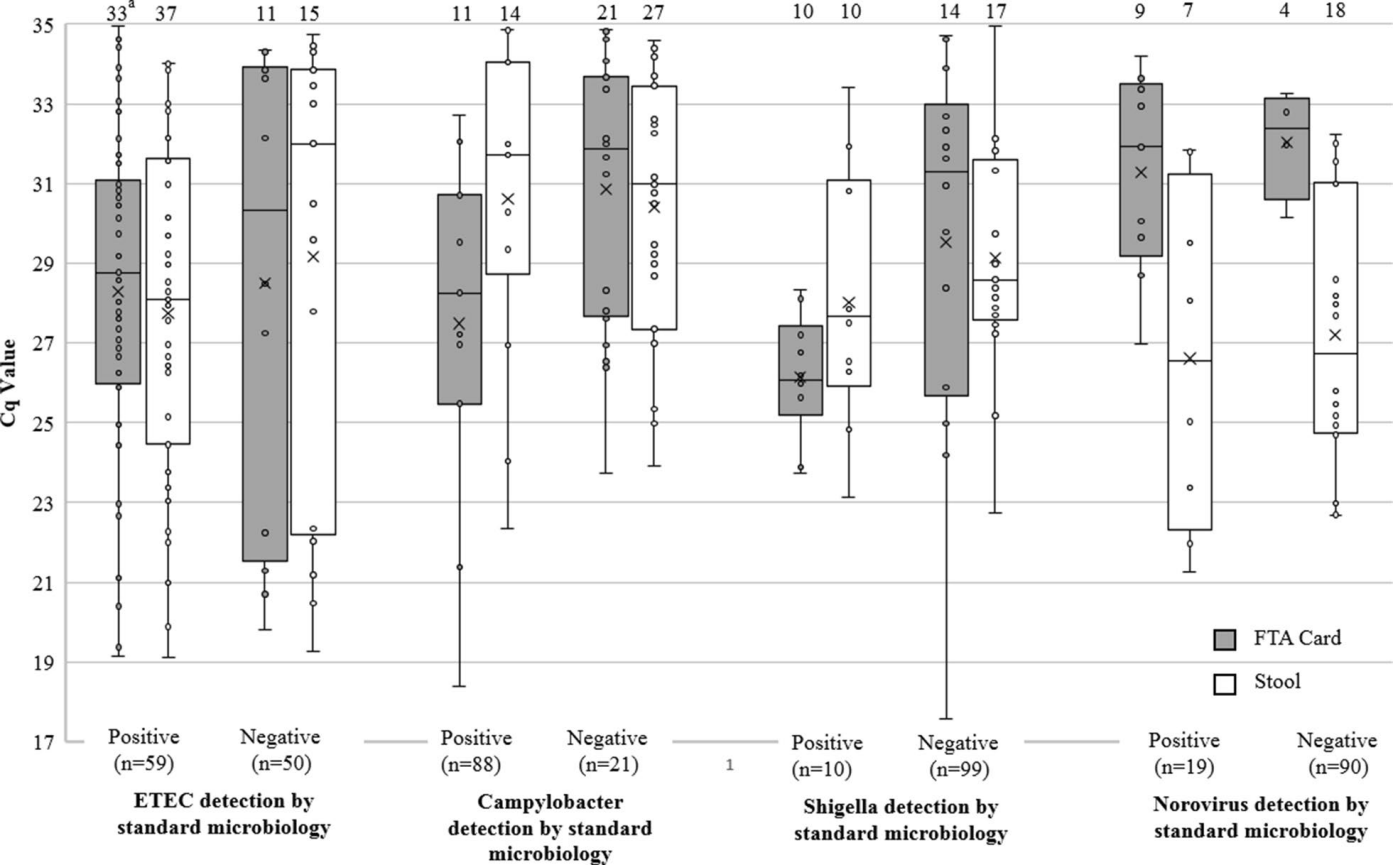
FTA card and stool samples positive vs. samples negative on microbiologic testing (n = 109). ETEC detection by TAC includes multiple targets: LT, STh and STp. Norovirus detection by TAC on FTA cards and stool includes the Norovirus GI or Norovirus GII target. Shigella and Campylobacter detection was based on a single target. Significant difference in median Cq values observed for the following by pairwise comparison: Campylobacter: Benchmark (+)/FTA Card(+) vs. Benchmark(−)/FTAcard(+) p = 0.036. Shigella/EIEC: Benchmark (+)/FTA Card(+) vs. Benchmark (−)/FTAcard(+) p = 0.046. Norovirus: Benchmark (−)/Stool (+) N = 18 Benchmark (-)/FTAcard(+) p = 0.003. Benchmark (+)/Stool (+) N = 7 Benchmark (+)/FTAcard(+) p = 0.033. The correlation between corresponding stool and FTA card targets was poor (r^2^ : ETEC 0.37, Campylobacter: 0.27, Shigella 0.40 and Norovirus 0.07; p < 0.01 for all)

Next, we compared Cq values of TAC on stool and FTA cards for 109 paired samples stratified by pathogen and results from benchmark testing (Fig. 2). We hypothesized that Cq values would be lower in FTA card and stool samples that were positive on benchmark testing vs. negative samples. A wide range of Cq values were observed across all pathogens and a significant difference in the median Cq value between benchmark positive and negative samples was only observed in FTA Cards positive for Campylobacter (p = 0.036) and Shigella (p = 0.046).

### Discussion

We tested archived stool specimens using TAC to determine the feasibility of using these specimens for clinical validation of TAC. The results show a lower than expected PPA of TAC on archival samples and significant variability in PPA estimates by pathogen when compared to previously published estimates [4, 5]. This is partly explained by the small sample size resulting in wide confidence intervals, an important limitation in our study as well as prior reports [7, 9]. The results suggest that using TAC on archival samples stored for several years may lower sensitivity and underestimate pathogen burden. Published reports, largely in the realm of microbiome research, focus on relatively short storage durations of days to months [11, 12]. Cannon et al. reported a 2.7% [95% CI 2.1–3.5%] decline in the percentage of samples positive on re-testing for norovirus by RT-PCR, with each additional year of storage at 4 °C and a—1 log loss of viral RNA titer with each 7-year period of sample storage at 4^○^C [13]. We were only able to detect 7 of 19 Norovirus positive stool samples (PPA: 36.8% [16.2–61.6]) despite a short duration of storage (2.6 years) at − 80^○^C. It is possible that the freeze–thaw cycles associated with transportation of specimens to the testing lab impacted viral recovery. Stool samples underwent two freeze thaw cycles since shipment of stool and FTA Cards could not occur simultaneously. Bacterial targets had higher TAC PPAs (approximately 65% for ETEC and Campylobacter) with Shigella/EIEC being the highest (10 of 10 samples positive by culture) despite a median storage duration of 7.6 years. The large variance in PPA estimates for bacterial targets and additional positives detected by TAC on FTA cards suggest that sampling error may contribute to an underestimation of the PPA, since pathogens may not be homogenously distributed in stool. Homogenization of stool samples prior to storage and the use of multiple samples from a specific time-point may reduce the variance due to sampling error [14]. In addition, careful documentation of processing, storage and sampling methods and an understanding of their impact on quantitative PCR data is needed to appropriately adjust detection estimates using archival samples.

We also evaluated smeared FTA Cards for storage and transportation of archival samples. Unfortunately, delays in receiving the stool specimens resulted in FTA Cards being stored for 2–3 years at room temperature prior to extraction and testing and negatively impacted PPA estimates for bacterial targets especially at higher stool Cqs. The PPA of TAC on FTA Cards across bacterial targets was lower than estimates from a post hoc analysis using fresh stool samples smeared on to FTA Cards and stored for approximately 2 years prior to extraction and testing [9]. This finding suggests that using archival samples that are freeze–thawed may negatively impact TAC performance on smeared FTA Cards compared to fresh stool. Additional studies are needed to evaluate strategies for increasing PCR yield from smeared FTA card such as reducing storage duration of FTA Cards, refrigeration or adding preservatives (e.g. RNAlater [Invitrogen, Carlsbad, CA]).

Our pilot study adds important insights into the use of repository samples to validate quantitative PCR assays. TAC sensitivity on archival specimens may be lower than previous estimates using specimens with a shorter duration of storage. The study also highlights the importance of developing standard operating procedures at the inception of studies to optimize processing and preservation of fecal samples and enhance the comparability and reproducibility of data [15, 16]. Future efforts should focus on adequately powered studies of TD stool archival specimens in order to understand the impact of storage duration on quantitative PCR data.

## Limitations

An important limitation of our study was the small sample size of bacterial targets. We could not evaluate the loss of PCR signal with increasing storage duration since TAC testing of stool samples and FTA cards occurred at a single time-point and not longitudinally at pre-specified time intervals. Microbiologic testing performed at the time of collection (i.e. benchmark) varied by site and time period, confounding the association between storage duration and TAC sensitivity. Unknown freeze-thaw cycles in archived specimens could also impact TAC performance on smeared FTA Cards compared to fresh stool.

## Data Availability

The datasets used and/or analyzed during the current study are available from the corresponding author on reasonable request.

## Abbreviations

TAC: TaqMan Array Card
TD: Travelers’ Diarrhea
PPA: Positive Percent Agreement
ETEC: Enterotoxigenic *E coli*
PCR: Polymerase Chain Reaction
Cq: Quantification cycle
DoD: Department of Defense
NAMRU-6: Naval Medical Research Unit-6, Lima, Peru
AFRIMS: Armed Forces Research Institute of Medical Sciences
NPA: Negative Percent Agreement
IRB: Institutional Review Boards
NMCP: Naval Medical Center Portsmouth
